# Synthesis of Imidazolium based PILs and Investigation of Their Blend Membranes for Gas Separation

**DOI:** 10.3390/membranes9120164

**Published:** 2019-12-03

**Authors:** Thanasis Chouliaras, Aristofanis Vollas, Theophilos Ioannides, Valadoula Deimede, Joannis Kallitsis

**Affiliations:** 1 Department of Chemistry, University of Patras, GR 26504 Patras, Greece; thxouliaras@yahoo.gr (T.C.); aristofanis.vollas@gmail.com (A.V.); kallitsi@upatras.gr (J.K.); 2 Foundation for Research and Technology, Institute of Chemical Engineering Sciences (FORTH/ICE-HT), GR 26504 Patras, Greece; theo@iceht.forth.gr

**Keywords:** PILs, blends, membranes, gas separation, pyridinium based PIL (PILPyr), imidazolium based PILs

## Abstract

Polymeric (ionic liquid) (PIL) copolymers bearing cationic imidazolium pendants and polar acrylic acid groups (P(VBCImY-co-AA_x_)), which both favor the interaction with CO_2_ molecules, have been synthesized and blended with film forming, high glass transition temperature aromatic polyether-based pyridinium PILs (PILPyr). The blend membranes based on the above combination have been prepared and characterized in respect to their thermal and morphological behavior as well as to their gas separation properties. The used copolymers and blends showed a wide range of glass transition temperatures from 32 to 286 °C, while blends exhibited two phase morphology despite the presence of polar groups in the blend components that could participate in specific interactions. Finally, the membranes were studied in terms of their gas separation behavior. It revealed that blend composition, counter anion type and acrylic acid molar percentage affect the gas separation properties. In particular, PILPyr-TFSI/P(VBCImTFSI-co-AA_20_) blend with 80/20 composition shows CO_2_ permeability of 7.00 Barrer and quite high selectivity of 103 for the CO_2_/CH_4_ gas pair. Even higher CO_2_/CH_4._ selectivity of 154 was achieved for PILPyr-BF_4_/P(VBCImBF_4_-co-AA_10_) blend with composition 70/30.

## 1. Introduction

Gas separation membranes are extensively applied in natural gas purification, CO_2_ capture, hydrogen recovery, and oxygen and nitrogen enrichment [1,2]. Among the various types of proposed membranes, polymeric membranes offer the advantages of energy efficiency, small footprint and scalability. Polymeric (ionic liquids) (PILs) have recently attracted considerable attention as CO_2_ separation membranes because they combine the inherent properties of polymers (e.g. processability, robustness) with the unique properties of ILs such as high CO_2_ sorption capacity, tunable physicochemical properties, and high thermal stability [3,4,5]. The majority of the PILs based on imidazolium, pyrrolidinium, or ammonium have been synthesized via free radical polymerization and show high CO_2_ permeability but low operation temperature and poor mechanical stability [6,7,8,9]. In order to overcome these obstacles and further increase the operation temperature, improve the separation properties and broaden their application range, the combination of a polymeric ionic liquid with a high glass transition temperature (T_g_) polymer seems to be an ideal solution [10,11,12]. The idea of using polymer blends for the development of gas separation membranes gives also additional freedom on the selection of the blend components in order to control the miscibility of phase separation. Phase behavior of blends plays a critical role in determining gas transport properties. In particular, miscible polymer blends display gas separation properties that are intermediate of their comprising individual polymers, while immiscible blends can lead to unique properties depending on the membrane morphology, volume fraction, size and shape of the dispersed phase [13,14,15]. The fundamental understanding of phase behavior and the resulting molecular interactions are essential for developing desirable membrane materials for gas separation. In recent years, membrane research in blends has largely focused on miscible polymer blends and on uncompatibilized immiscible blends, which reflects the need for higher gas throughput.

Several blended membranes for gas separation have been reported so far, such as Matrimid/polybenzimidazole [16,17], polyimide/polysulfone [18] and polyimide/polybenzimidazole [19,20]. As an example, polybenzimidazole has been used in blends with several, low gas permeability types of polyimide (matrimide, torlon and P84) [16], where both polymers were selected due to their high thermal, chemical and mechanical resistance. In addition, polybenzimidazole (PBI) has very low permeation properties but high selectivity for various gas pairs. Blends that were prepared at different ratios, showed a selectivity increase for different gas pairs and a drop-in gas permeability that was correlated with PBI concentration in the blend. 

Additionally, aromatic polyether based pyridinium PILs for use as CO_2_/CH_4_ gas separation membranes have been developed [10] that show high glass transition temperature, excellent thermal stability, enhanced solubility and outstanding film forming ability [21,22,23,24,25]. Since the CO_2_ permeability at ambient temperature is very low but their selectivity for the CO_2_/CH_4_ gas pair is quite favorable (with values up to ~50), a further increase of CO_2_ permeability while at the same time maintaining the satisfactory CO_2_/CH_4_ selectivity, could make these membranes promising candidates for natural gas and biogas purification.

In this study, we have focused on the above mentioned aromatic polyethers due to their superior solubility combined with high glass transition temperatures and a series of N-methyl imidazolium containing poly(vinyl benzyl chloride-co-acrylic acid) backbone PILs that were chosen and synthesized as the second component of blend materials. In particular, bulky imidazolium pendant groups can increase gas permeability due to chain packing disruption as well as due to their improved CO_2_ solubility. The incorporation of polar carboxylic acid groups onto the polymeric backbone also aims at the improvement of CO_2_ solubility. Thus, blends comprised of high molecular weight, thermally stable pyridinium based PILs (PILPyr) with excellent film forming ability and imidazolium functionalized poly(vinyl benzyl chloride-co-acrylic acid) backbone PILs have been prepared at different compositions. The effects of blend composition on miscibility, microstructure and gas separation performance of membranes are also investigated.

To the best of our knowledge, this is the first study focusing on the development of membranes by blending two different types of PILs for natural gas purification.

## 2. Materials and Methods 

### 2.1. Materials

Aromatic polyethers bearing main chain pyridine units (M_n_ 60,000), were synthesized according to a previously reported procedure [20]. Thus, 4-vinylbenzyl chloride (VBC, 90%), acrylic acid (AA, 99%), lithium bis(trifluoromethyl)sulfonamide (TFSI, ≥99%), 1-methylimidazole (99%), dimethyl sulfate (DMSA, ≥ 99%), azobisisobutyronitrile (AIBN) (98%) and ethyl acetate (99.5%) were purchased from Sigma-Aldrich, Steinheim, Germany. Dimethylformamide (DMF, anhydrous, 99.8%), dimethylacetamide (DMAc, ≥99%), dimethylsulfoxide (DMSO, ≥99.7%), chloroform (99.8+%), diethyl ether (99%) and sodium tetrafluoroborate (NaBF_4_, 97%) were supplied from Fischer, Pittsburgh, Pennsylvania, WI, USA. All solvents and chemicals were used as received. Single gases, CO_2_, CH_4_, He, Ar were supplied by Air Liquide, Paris, France with 99.99% minimum purity.

### 2.2. PIL Synthesis

#### 2.2.1. Synthesis of Pyridinium-Based PILs (PILPyr)

Pyridinium based PILs (PILPyr) have been synthesized by modification of aromatic polyether copolymers precursors bearing main chain pyridine units, as reported very recently by our group [10]. Specifically, precursor copolymers were converted to their PIL analogues via N-methylation and anion exchange metathesis reaction with the desirable anions (tetrafluoroborate and bis(trifluoromethylsolfonyl)imide). High molecular weight precursor copolymers ((M_n_ = 62,000, M_w_ = 128,000, PDI = 2.1) were prepared through nucleophilic aromatic substitution step growth polymerization of the tetramethyl biphenyl diol with 2,5 diphenyl substituted pyridine diol (molar ratio 60/40) and diphenyl sulfone difluoride, as described elsewhere [21]. 

#### 2.2.2. Synthesis of Imidazolium-Based PILs (P(VBCImY-co-AA_x_))

##### Synthesis of Precursor Copolymers P(VBC-co-AA_x_) 

The synthesis of poly(4-vinyl benzyl chloride-co-acrylic acid) precursor copolymers has been described previously by our group [26,27]. The copolymers were prepared via conventional free radical polymerization copolymerization of 4-vinyl benzyl chloride and acrylic acid (AA) monomers using azobisisobutyronitrile (AIBN) as an initiator and dimethylformamide (DMF) as solvent at 70 °C for 24 h. Copolymers with different compositions were prepared by varying the molar feeding ratios (90/10, 80/20). These copolymers will be denoted as P(VBC-co-AA_x_) copolymers where *x* is the molar fraction of AA units as determined by the Proton Nuclear Magnetic Resonance spectroscopy (^1^HNMR) characterization in CDCl_3_.

##### Synthesis of Imidazolium Functionalized P(VBCImCl-co-AA_x_) 

The N-quaternization reaction of P(VBC-co-AA_x_) copolymers is described as follows: 2 g of P(VBC-co-AA_20_) (14.65 mmol) and 8 mL of DMF were added under argon atmosphere in a degassed round bottom flask. After the complete dissolution of the copolymer, 1.40 mL of 1-methylimidazole (17.58 mmol) was added to the yellowish solution. The reaction temperature was raised to 70 °C and the reaction mixture was left stirring for 24 h. One hour after the addition of 1-methylimidazole, a white solid started to precipitate and was dissolved by adding 2 mL of methanol. The quaternized copolymer was precipitated in diethyl ether, filtered and washed several times with diethyl ether. The obtained white powder was purified by re-dissolving in methanol and precipitation in diethyl ether. The product, denoted as P(VBCImCl-co-AA_20_), was dried in a vacuum oven for 12 h at 40 °C. 

##### Anion Exchange of Imidazolium Functionalized P(VBCImCl-co-AA_x_) 

Imidazolium based PILs were obtained by exchanging the chloride anion of the P(VBCImCl-co-AA_x_) with BF_4_^−^ or TFSI^−^ anions, using NaBF_4_ or LiTFSI salts respectively. In a typical anion exchange process (taking TFSI^−^ as an example), 3 g of P(VBCImCl-co-AA_20_) were dissolved in 30 mL DMSO. A solution of LiTFSI in DMSO (1.5 molar equivalents of LiTFSI salt) was added dropwise into the copolymer solution. The reaction mixture was stirred for 48 h at room temperature. The resulting PIL, denoted as P(VBCImTFSI-co-AA_20_), was obtained by precipitation in deionized water. The solid white product was washed with deionized water, filtered and dried in vacuum oven at 40 °C for 12 h. The same procedure was followed for BF_4_^−^ using NaBF_4_ salt (The PIL denoted as P(VBCImBF_4_-co-AA_20_)).

### 2.3. Membrane Preparation

Neat pyridinium and imidazolium based PILs as well as their blends (PILPyr/P(VBCImY-co-AA_x_) were examined regarding their film forming ability using the solution casting method. For pure PILs, 3 and 13 wt% solutions were prepared in DMA for PILPyr and P(VBCImY-co-AA_x_), respectively. The former was poured on a glass surface while the later was poured onto a Teflon surface, both left at 80 °C for 24 h to evaporate slowly the solvent. In the case of the blends, the two PILs components were initially dissolved separately (3% w/v in DMA, each solution) and then mixed together in different weight ratios to prepare a homogeneous solution. The obtained solution was casted on a flat glass surface at 80 °C for 24 h. After solvent evaporation, the formed membranes were peeled off and dried in a vacuum oven at 80 °C until constant weight was achieved. 

The thickness of neat PILs and blend membranes (40–50 μm) was estimated using SEM microscopy, where average thickness was calculated from six measurements taken at different locations in each membrane sample.

### 2.4. Characterization

The ^1^HNMR and Fluorine Nuclear Magnetic Resonance (^19^FNMR) spectra were recorded on an Advance DPX 400 MHz spectrometer (Bruker, Karlsruhe, Germany). All solutions were made either in deuterated chloroform (CDCl_3_) or dimethylsulfoxide (DMSO-d_6_) and tetramethylsilane (TMS) was used as internal standard.

Thermogravimetric analysis (TGA) was conducted in the temperature range from room temperature to 800 °C under nitrogen atmosphere and a heating rate 20 °C min^−1^ using a Labsys TG (Setaram Instrumentation, Caluire-et-Cuire, France). 

Attenuated Total Reflection Fourier Transform Infra Red (ATR-FT-IR) spectra were collected on a Platinum ATR spectrometer (Bruker, Ettlingen, Germany).

Scanning Electron Microscopy (SEM) was conducted on a LEO Supra 35VP microscope (Zeiss, Oberkochen, Germany). Membrane cross-sections for SEM imaging were prepared by freeze-fracturing the samples after immersion in liquid nitrogen.

Differential Scanning Calorimetry (DSC) was carried out on a Perkin Elmer DSC instrument (DSC Q100, Waltham, MA, USA) from −100 to 300 °C with a heating rate of 10 °C/min under a nitrogen flow. The effect of water and previous thermal history was erased by the first heating run while the glass transition temperatures were recorded in the second heating run.

### 2.5. Gas Separation Experiments

The room-temperature permeation of CO_2_ and CH_4_ pure gases was examined by the Wicke-Kallenbach method [28]. The membrane samples were disc-shaped with 5 cm diameter. The permeate side of the cell was purged with helium flowing at a rate of 20 cm^3^ min^−1^ and a pressure of 1 atm. Analysis of CH_4_ and CO_2_ molar fraction in the helium permeate stream was performed by gas chromatography (Shimadzu GC-2014, Kyoto, Japan, equipped with thermal conductivity (TCD) and flame ionization (FID) detectors and Porapak Q and Carboxen chromatographic columns). 

A mass transfer mechanism incorporating solution and diffusion processes is generally used to describe the transport of gases through a dense polymeric membrane [29]. The permeability (*P*) can be calculated from Equation (1) [1,30]:

(1) $$P=\frac{J}{\Delta p}l$$

where *P* is the permeability in Barrer units and *Δp* = *p*_1_ – *p*_2_, *p*_1_ and *p*_2_ being the partial pressures of the probe gas in the feed and permeate side (cm Hg), respectively. *J* represents the steady-state flux (cm^3^/cm^2^ s) and *l* is the thickness of the membrane (cm).

Three different membranes prepared under identical conditions were employed in the permeation measurements to confirm the reproducibility of the preparation method. The ideal selectivity, *α*(CO_2_/CH_4_), of the CO_2_/CH_4_ pair was calculated by the following equation:

(2) $$\alpha \left({\mathrm{CO}}_{2}/{\mathrm{CH}}_{4}\right)=\frac{P_{\mathrm{CO}2}}{P_{\mathrm{CH}4}}$$

## 3. Results and Discussion

### 3.1. Synthesis and Characterization of Imidazolium-Based PILs

Combination of two polymeric ionic liquids obtained from chain growth (P(VBCImY-co-AA*_X_*)) and step growth (pyridinium based PIL, PILPyr) polymerizations were attempted here in order to take advantage of the high ionic liquid content and the presence of CO_2_ interactive groups like the carboxy groups in the P(VBCImY-co-AA*_X_*) as well as of the excellent film forming properties and the high T_g_ of pyridinium based PILs. Thus, pyridinium based PILs have been synthesized via modification of robust, high T_g_, aromatic polyethers bearing main chain pyridine units [10]. The prepared PILs showed excellent film forming ability-free standing membranes with thickness down to 3 μm were fabricated—nevertheless, low CO_2_ permeability was obtained due to the close polymer chain packing. One way to increase CO_2_ permeability is to disrupt dense polymer chain packing by blending with another polymer containing bulky, CO_2_-philic groups. Thus, PIL copolymers bearing cationic imidazolium pendants and polar acrylic acid groups, which both favor the interaction with CO_2_ molecules, have been synthesized in order to be used as blend constituents. So, the first objective was to investigate the effect of blend composition on gas transport properties of the prepared blend membranes. Moreover, a second objective was to study whether the incorporation of polar acrylic acid groups into the imidazolium based PILs would provide better miscibility with pyridinium based PILs in blend membranes.

**Scheme 1:**
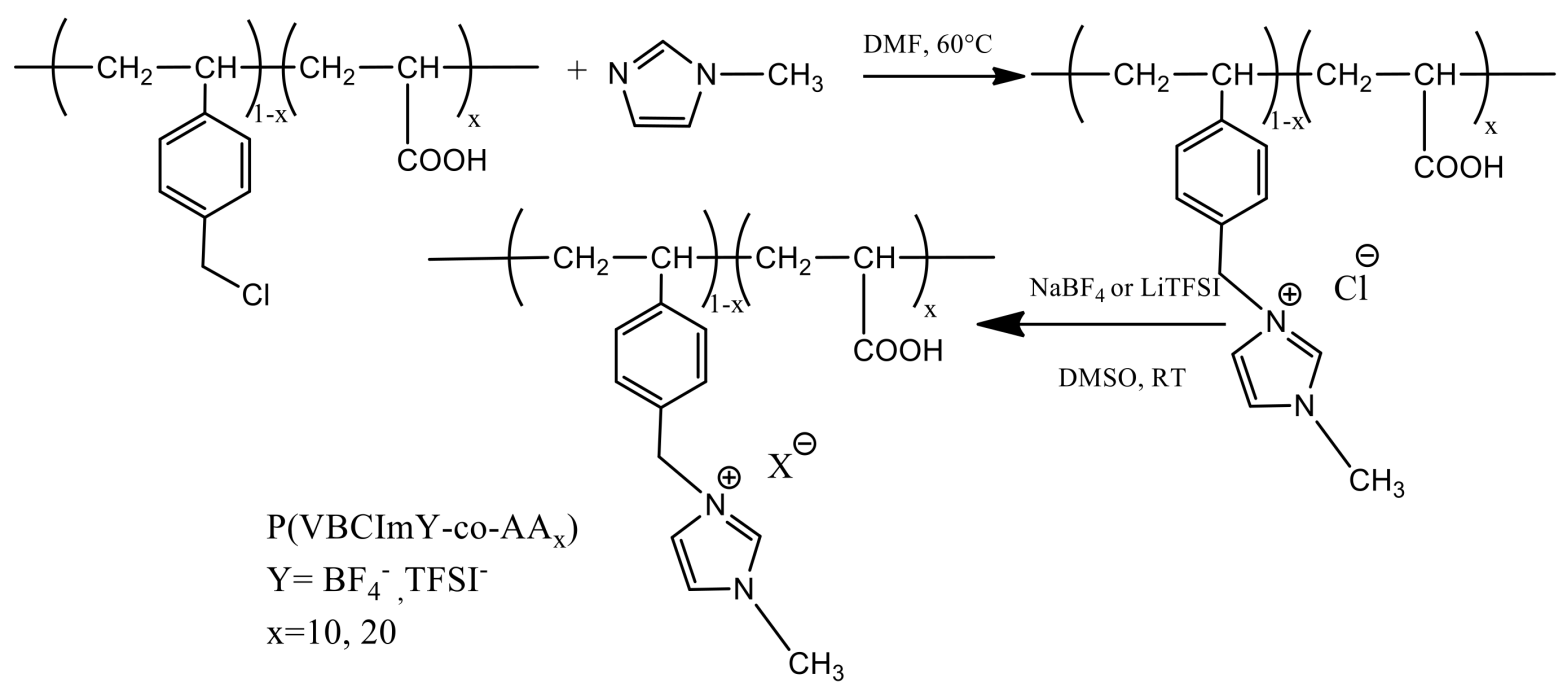
Synthetic route for synthesis of P(VBCImY-co-AA_x_) based PILs.

Imidazolium based PILs have been synthesized via the modification of a precursor copolymer containing vinyl benzyl chloride and acrylic acid units (P(VBC-co-AA_x_) as described in detail elsewhere [26,27]. The same polymeric backbone was also chosen for the synthesis of pyrrolidinium based PILs to be used as dye adsorbents [31]. By varying the molar ratios of vinyl benzyl chloride and acrylic acid monomers, copolymers with different compositions were obtained, as verified by ^1^HNMR spectroscopy. Although different experimental conditions (e.g. reduction of initiator/monomer molar ratio, increase of monomer concentration) were followed in order to obtain high molecular weight copolymers, in all cases, relatively low molecular weight copolymers (M_w_ = 12,000–17,000) were obtained, as determined using gel permeation chromatography (GPC). The corresponding imidazolium-based copolymers were synthesized via post modification of P(VBC-co-AA_x_) precursors through quaternization reaction of 1 methyl-imidazole with reactive benzyl chloride moieties. The PILs were converted to the desired anion form after anion exchange reaction with LITFSI or NaBF_4_ salts. The general synthetic route of imidazolium based PILs is illustrated in Scheme 1.

For reasons of simplicity, copolymers are denoted as P(VBCImY-co-AA*_X_*) where **Y** = TFSI, BF_4_, refers to anions, while *X* refers to the molar percentage of acrylic acid (AA) groups. The successful synthesis of the PILs was confirmed via proton nuclear magnetic resonance.

**Figure 1:**
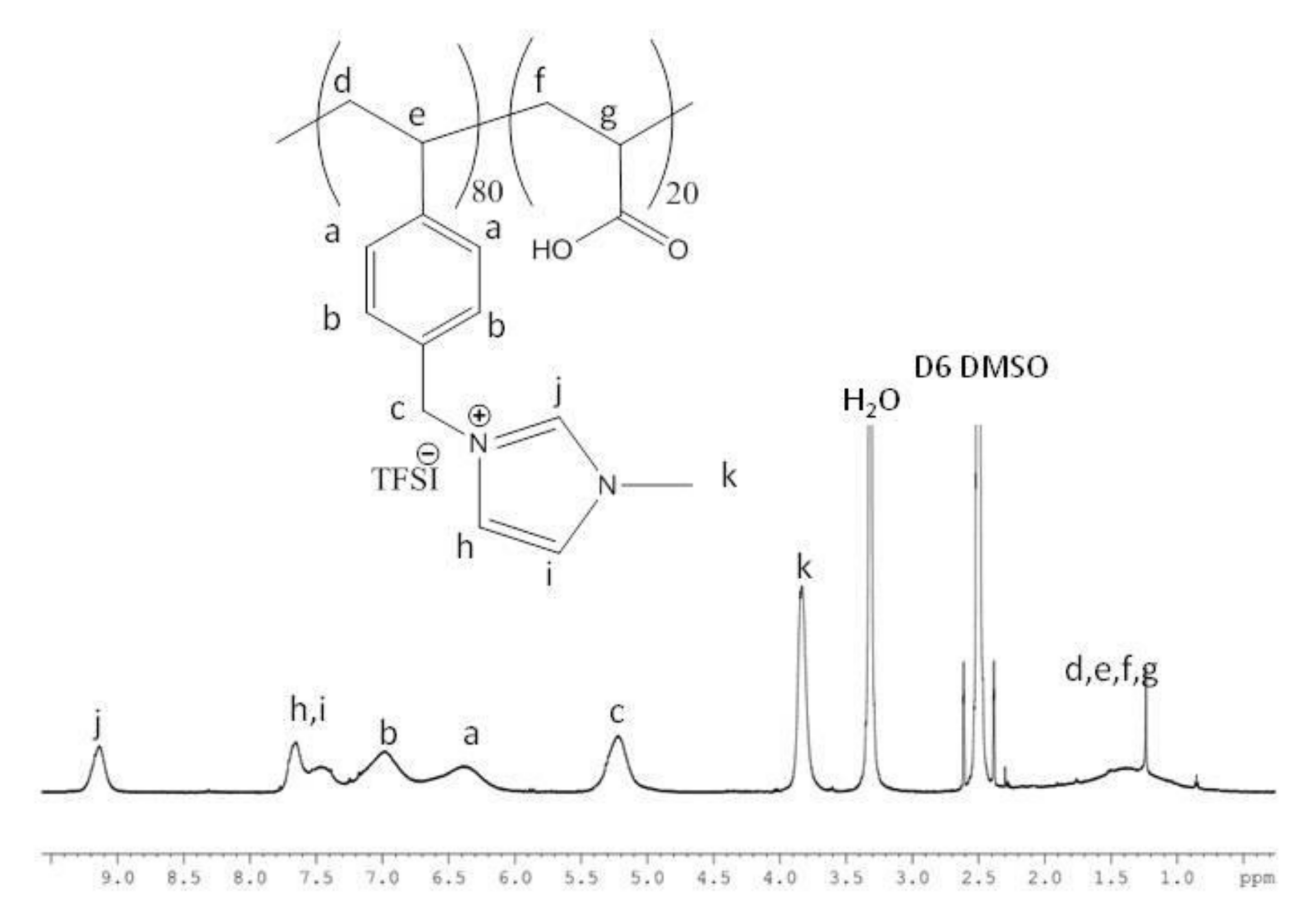
Proton Nuclear Magnetic Resonance (^1^HNMR) spectrum of imidazolium functionalized copolymer P(VBCImTFSI-co-AA_20_) in deuterated dimethylsulfoxide (d_6_-DMSO).

A characteristic ^1^HNMR spectrum of P(VBCImTFSI-co-AA_20_) based PIL with bis(tri-fluoromethylsulfonyl) imide as anion is given in Figure 1. The ^1^HNMR spectra of the precursor copolymer P(VBC-co-AA_20_), the P(VBCImCl-co-AA_20_) PIL as well as the P(VBCImBF_4_-co-AA_10_) based PIL with tetrafluoroborate as anion are given in Figure S1. 

In the ^1^HNMR spectrum, the broad peaks in the range 0.8–2.1 ppm are assigned to the backbone protons of both poly(acrylic acid) (PAA) and PIL components. The CH_2_–N methylene protons **c**, close to the positively charged nitrogen are located at 5.2 ppm while the signals at 9.1 ppm and in the range 7.3–7.7 ppm are assigned to the imidazolium ring methine protons j and i, h protons, respectively. The broad peaks in the 6.3–7.3 ppm region correspond to the aromatic protons (a, b) of VBC backbone. All these data confirm the successful quaternization reaction. Moreover, evidence for the successful exchange to TFSI^−^ anions was collected by ^1^HNMR and ^19^F NMR spectroscopy (ESI, Figures S1 and S2). Regarding the ^19^F NMR spectrum of P(VBCImTFSI-co-AA_20_), a strong peak appears at −78.74 ppm which is assigned to the CF_3_ group of TFSI^−^ anion [12].

**Figure 2:**
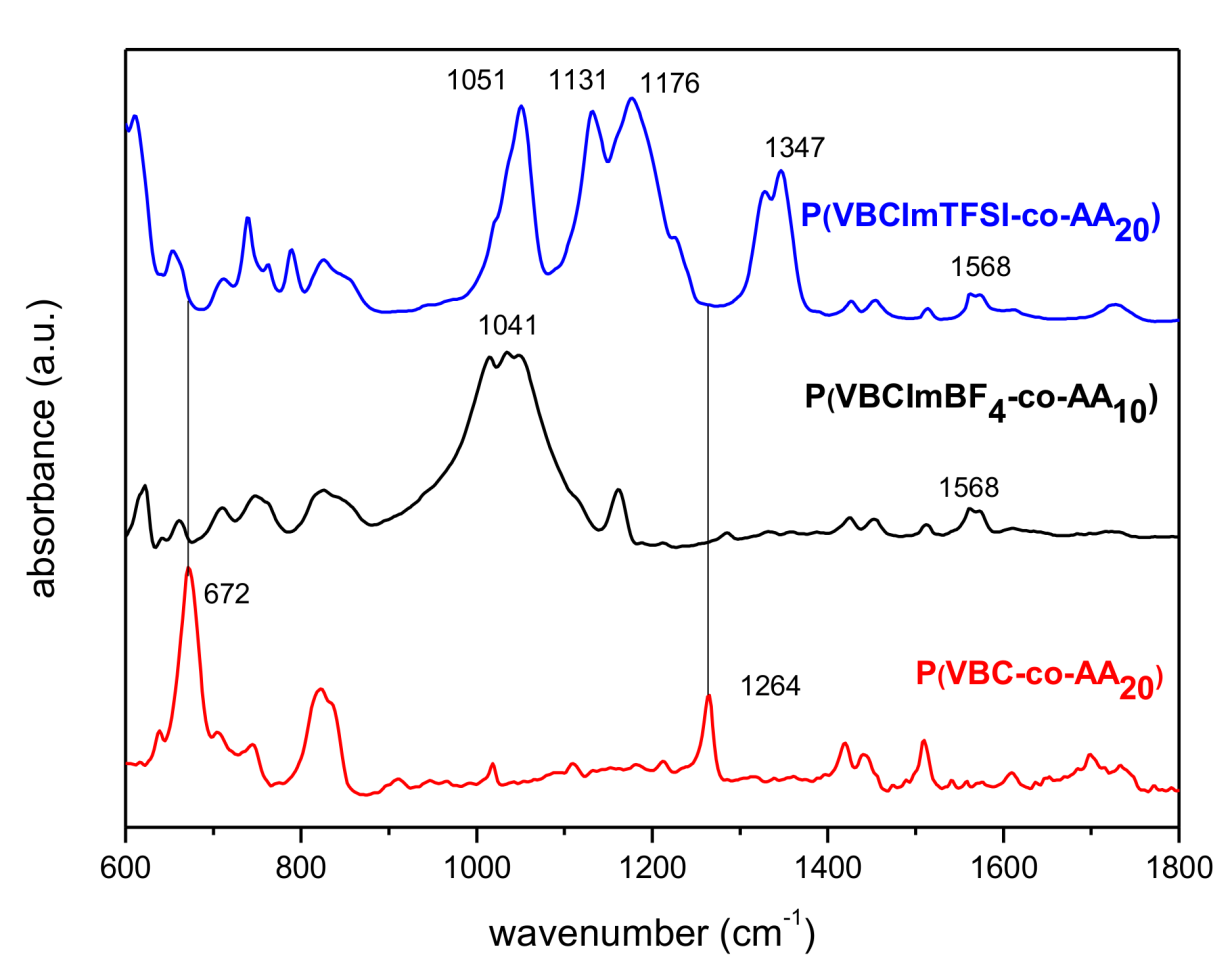
Attenuated Total Reflection Fourier Transform Infrared (ATR–FT–IR) spectra of precursor copolymer P(VBC-co-AA_20_), P(VBCImBF_4_-co-AA_10_) and P(VBCImTFSI-co-AA_20_).

FT-IR spectroscopy was also used to verify the chemical structure of the synthesized PILs. Comparison of the FT–IR spectra of precursor P(VBC-co-AA_20_) copolymer, P(VBCImTFSI-co-AA_20_) and P(VBCImBF_4_-co-AA_10_) (Figure 2) reveals the disappearance of the peaks at 1264 and 672 cm^−1^ corresponding to stretching of the C-Cl bond and bending of CH_2_–Cl group of precursor P(VBC-co-AA_20_) after quaternization reaction. In addition, a new peak emerges at 1568 cm^−1^ assigned to the C=N bond of the imidazolium group [32,33].

In the case of P(VBCImTFSI-co-AA_20_), the pronounced peak located at 1176 cm^−1^ is assigned to C–F stretching of the CF_3_ group and the bands at 1347 and 1131 cm^−1^ can be attributed to the stretching of S–O in SO_2_ group. Finally, the asymmetric stretching band of the S–N–S group (1051 cm^−1^) can be identified as well, confirming the presence of the TFSI counter anion [34]. In P(VBCImBF_4_-co-AA_10_), the new band centered at 1041 cm^−1^ is assigned to the B-F stretching vibration [35].

**Figure 3:**
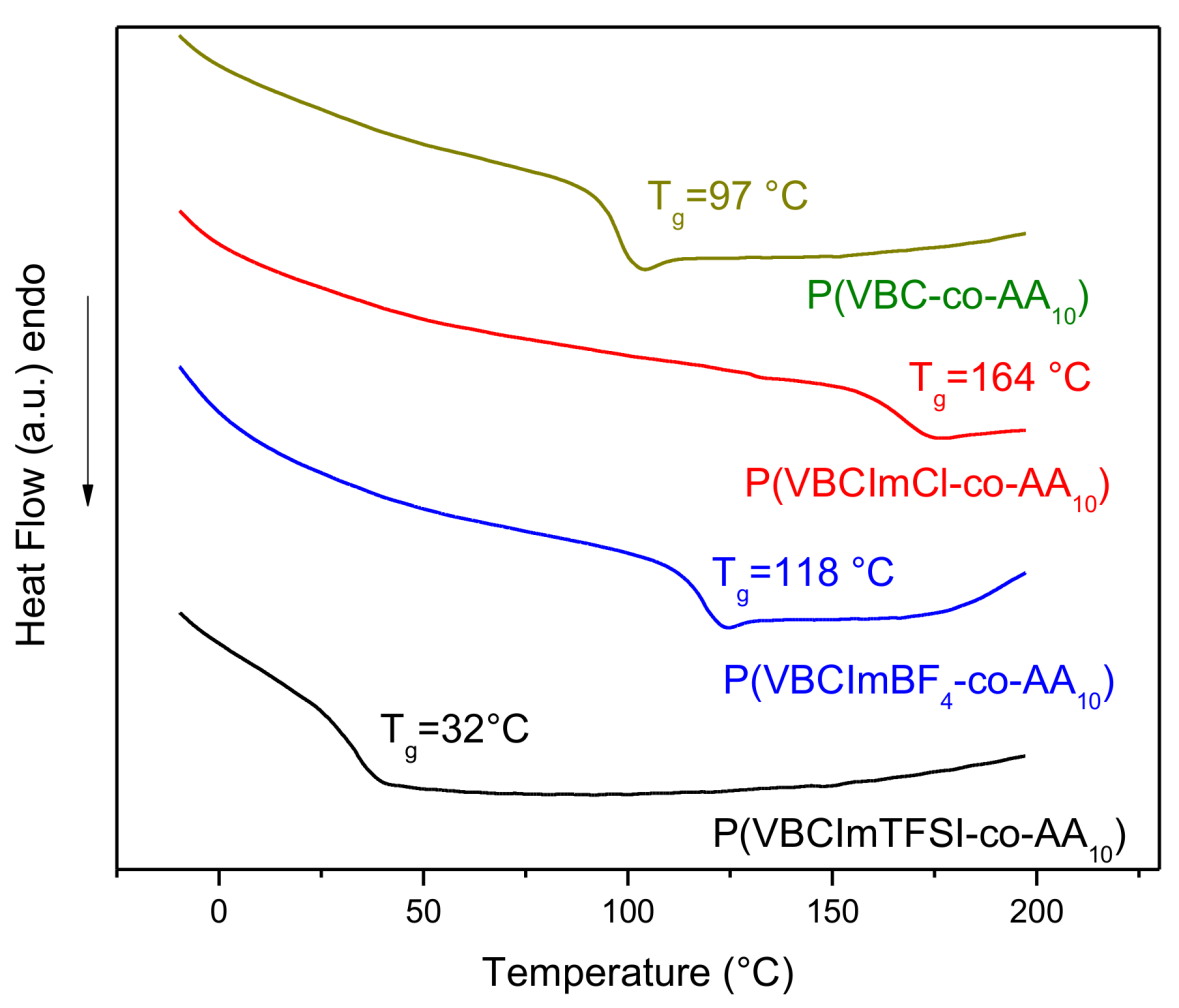
Differential scanning Calorimetry (DSC) thermograms of precursor copolymer P(VBC-co-AA_10_) and its corresponding PILs with different counter anions.

Differential scanning Calorimetry (DSC) was used to determine glass transition temperatures of the synthesized P(VBCImY-co-AA_x_) based PILs. The modification of the P(VBC-co-AA_10_) precursor to its PIL analogue containing the Cl^−^ anion resulted in a noticeable increase of T_g_ from 97 to 164 °C which could be probably attributed to the strong hydrogen bond formation between the chloride anion and the C-2 position proton of imidazolium group [34] (Figure 3). On the other hand, in the case of TFSI and BF_4_ anions, T_g_ is much reduced when compared to PIL containing the Cl anion, as these anions cannot form strong hydrogen bonds. P(VBCImTFSI-co-AA_10_) with TFSI anion is soft and has the lowest T_g_ value (32 °C) in comparison to BF_4_ (T_g_ = 118 °C) due to the presence of the bulkier TFSI anion which disrupts polymer chain packing density. In addition, the decreased T_g_ value is related to TFSI weakly coordinating anion nature which forms weak hydrogen bonds with C-2 proton of imidazolium group, facilitating thus polymer chain mobility [36].

Thermogravimetric analysis (TGA) was also used to evaluate the thermal stability of synthesized PILs (Figure S3). P(VBCImTFSI-co-AA_10_) showed increased thermal stability compared to the corresponding PILs with BF_4_ and Cl anions and its precursor. The PIL containing Cl anions showed a weight loss of 10% at temperature up to 150 °C due to traces of absorbed water since Cl anions are much more hydrophilic compared to the corresponding PILs containing the TFSI or BF_4_ anions. P(VBCImTFSI-co-AA_10_) started to decompose at 365 °C (defined as 5 wt% weight loss) while P(VBCImBF_4_-co-AA_10_) at 309 °C. The increased thermal stability of PIL containing the TFSI anion could be attributed to the reduced basicity of TFSI anion against BF_4_ and this behavior is in agreement with other studies [10,37].

### 3.2. Preparation and Characterization of PIL Blend Membranes

**Scheme 2:**
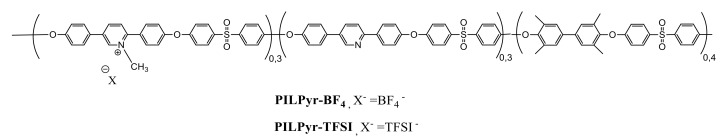
Chemical structure of pyridinium based PIL (PILPyr-X where X = BF_4_, TFSI anions).

The neat imidazolium based PIL membranes were quite brittle due to their low molecular weights, and, as a result, could not be directly used for gas permeability measurements. So, these PILs were blended with high molecular weight pyridinium based PILs (PILPyr, Scheme 2) in order to prepare robust blend membranes. In Table 1 are given the blend membranes prepared by varying blend composition, counter anion type and molar fraction (*X*) of acrylic acid units containing in P(VBCImY-co-AA*_X_*).

Thermal transitions of the neat PILs and blended films were investigated to probe the influence of blend composition on the phase structure of the blends, using BF_4_ as counter anion in both PIL blend constituents and having the molar fraction of acrylic acid units 10% as representative examples. DSC thermograms of PILPyr-BF_4_/P(VBCImBF_4_-co-AA_10_) blends are illustrated in Figure S4. Blend membranes show two T_g_s, suggesting that these blends are phase separated. Due to the small entropy of mixing, two mixed phases with differing composition are expected. The T_g_ at ~284 °C corresponds to the PILPyr-BF_4_ rich phase whereas the one at lower temperature corresponds to the P(VBCImBF_4_-co-AA_10_) rich phase.

Regarding the thermal stability of the prepared blends, the TGA curves of PILPyr-TFSI/ P(VBCImTFSI-co-AA_10_) blends are illustrated in Figure S5 as representative examples. Neat PILPyr-TFSI exhibited an early mass loss which is most likely due to residual water and DMA solvent (up to 205 °C has a weight loss 4.3%) followed by a single weight loss step with an onset degradation temperature at 240 °C. Blends 70/30 and 80/20 showed improved thermal stability and blend 80/20 had a small loss from solvent (3% weight loss around 212 °C) followed by a more significant onset of degradation around 280 °C. 

**Figure 4:**
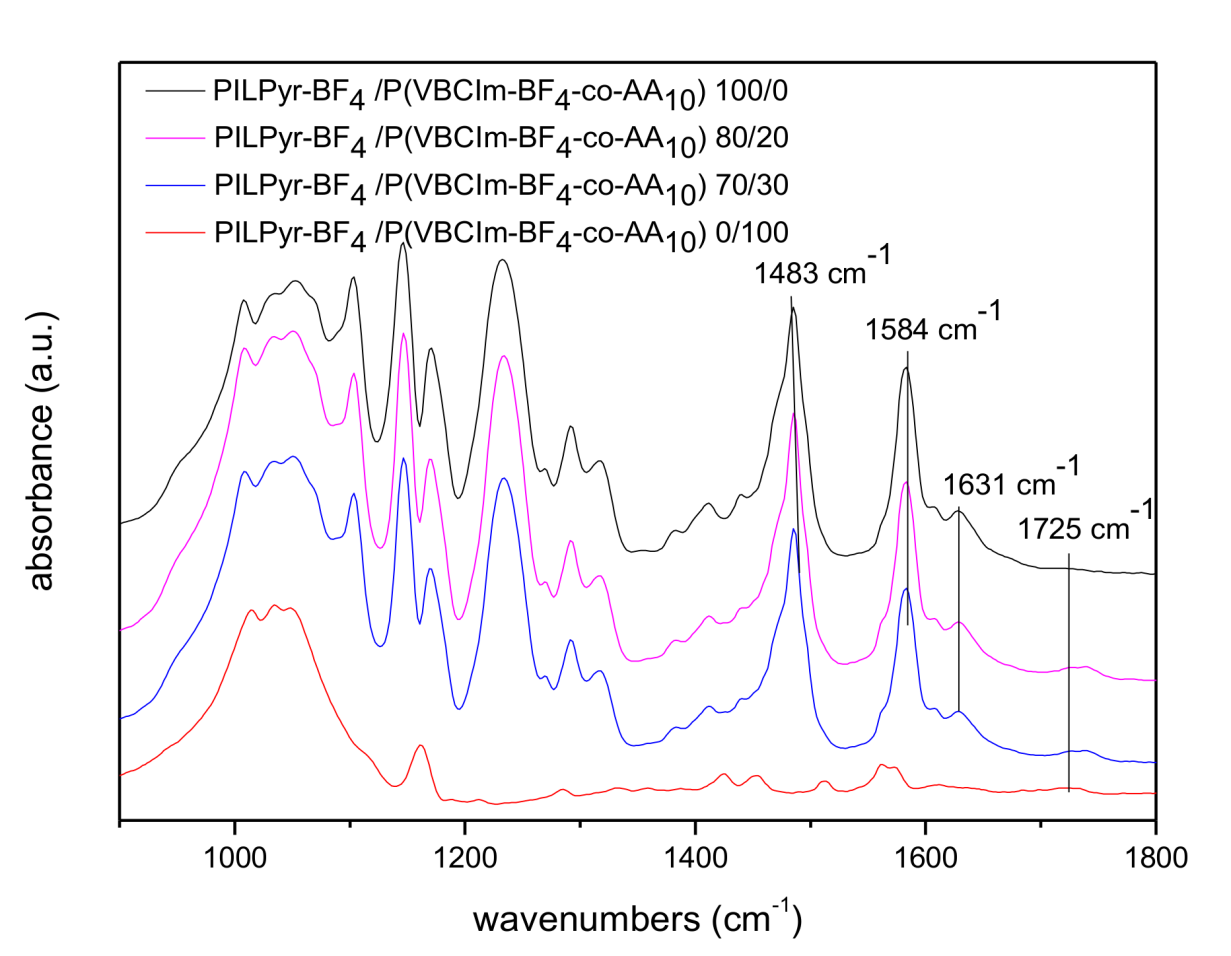
Attenuated Total Reflection Fourier Transform Infrared (ATR–FT–IR) spectra of PILPyr-BF_4_/P(VBCImBF_4_-co-AA_10_) blends.

The phase separated behavior of blends is further confirmed by the ATR–FT–IR and SEM data. ATR–FT–IR spectra of blend membranes confirm the presence of the characteristic peaks of both PILs (Figure 4). The neat PILPyr contains both pyridine and pyridinium units as only 50% N-quaternization degree was accomplished via conversion of the precursor aromatic polyether to its ionic analogue. Thus, characteristic peaks of both pyridine and pyridinium groups were observed. In specific, the bands centered at 1483 and 1631 cm^−1^ correspond to the stretching mode of pyridinium cation while the peak at 1584 cm^−1^ is attributed to the pyridine ring [38,39]. The shoulder located at 1609 cm^−1^ could probably be assigned to the free pyridine and pyridinium cation interaction. In the case of PILPyr-BF_4_/P(VBCImBF_4_-co-AA_10_) blends, with increasing P(VBCImBF_4_-co-AA_10_) composition, the peak at 1584 cm^−1^ remains unaltered, suggesting that free pyridine units do not participate in any interaction. Moreover, the intensity of the peaks at 1483 and 1631 cm^−1^ corresponding to pyridinium has also not changed upon blending. A closer inspection of neat P(VBCImBF_4_-co-AA_10_) in the region of 1700–1800 cm^−1^, reveals a broad peak located at 1725 cm^−1^, which can be attributed to the overlapping of two carbonyl stretching bands comprising of free as well as self-associated carboxylic groups [38]. It seems that this peak is shifted towards higher wavenumbers upon blending due to the liberation of carbonyl groups since hydroxyl groups of carboxylic acids probably participate to hydrogen bond formation with other groups (e.g. BF_4_ anion). It should be noted that these blends constitute a very complex system, making it difficult to study the development of specific electrostatic interactions between pyridine/pyridinium, pyridine/carboxylic acid, BF_4_/carboxylic acid groups. However, the absence of peak shifts or new peaks corresponding to hydrogen bonding formation between pyridine and carboxylic acid groups or protonation of pyridine by carboxylic acid groups (acid-base interaction) compared to pure PILs, implies that there are no strong interactions between the two PILs that could promote miscibility. The same trends were also observed in the case of PILPyr-TFSI/P(VBCImTFSI-co-AA_10_) blends where TFSI was used as counter anion in both PILs.

**Figure 5:**
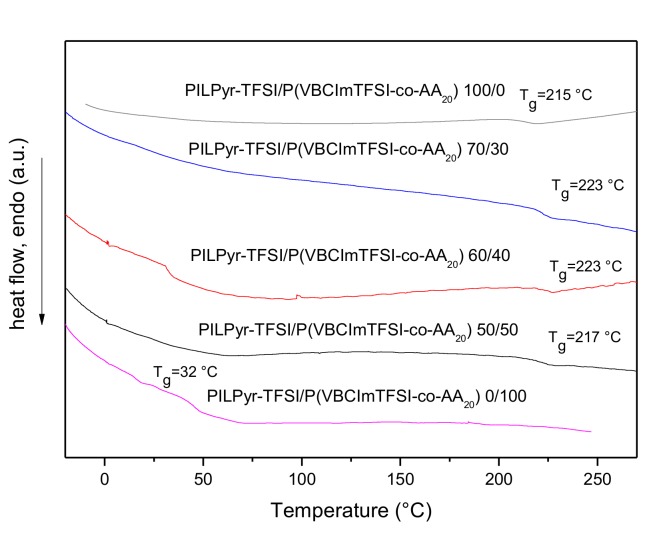
DSC thermograms of PILPyr-TFSI/P(VBCImTFSI-co-AA_20_) blends.

Since it is very important to prepare homogeneous, dense blend membranes for gas separation, one way to promote miscibility is to increase the number of acrylic acid units from 10 to 20% which can participate in specific interactions (e.g. hydrogen bonding) with polar free pyridine groups contained in PILPyr. Thus, PILPyr-TFSI/P(VBCImTFSI-co-AA_20_) blends with 20% molar percentage of acrylic acid units were studied with ATR-FT-IR spectroscopy, where no shift of new peaks was observed upon blending that could be attributed to specific interactions (Figure S6). These data are further supported by the DSC evidence where two peaks corresponding to the T_g_s of the pure blend components were revealed implying that these blends are also phase separated (Figure 5).

**Figure 6:**
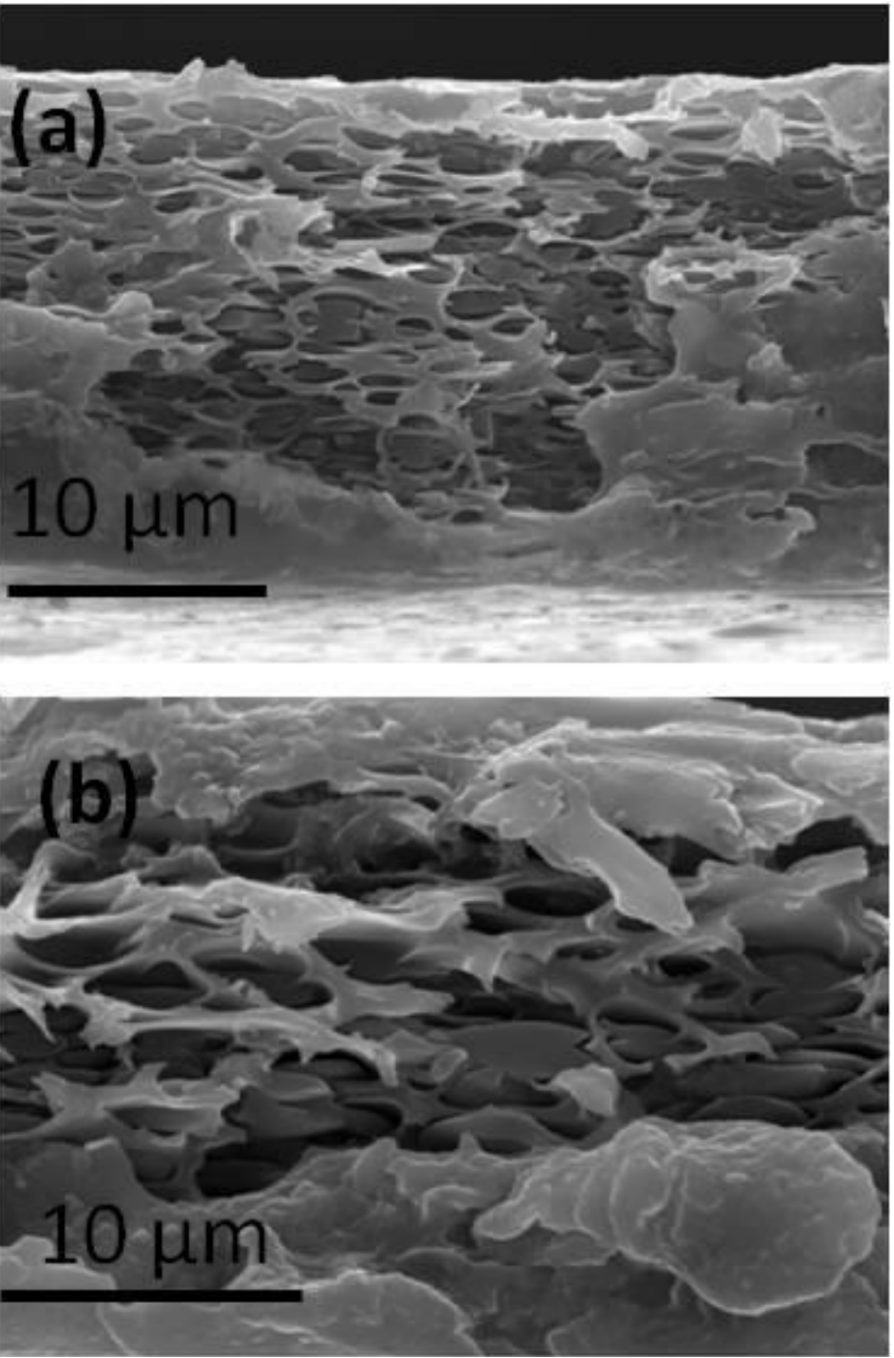
SEM micrographs of PILPyr-BF_4_/P(VBCImBF_4_-co-AA_10_) blend membranes (**a**) with composition 80/20 and (**b**) with composition 70/30.

The morphology of the blend membranes was studied by SEM microscopy. All blends exhibited biphasic morphology. Most of the two-phase blends consist of a continuous, single component phase into which a second component is dispersed. Typically, phase separated polymer blends can exhibit co-continuous, matrix-droplet, fiber and lamellar morphologies [40]. Morphology is highly dependent on the polymer blend composition. Figure 6 shows the cross-section SEM images of PILPyr-BF_4_/ P(VBCImBF_4_-co-AA_10_) blend membranes (having the BF_4_ anion in both blend components) containing 20 and 30 wt% of P(VBCImBF_4_-co-AA_10_). It should be mentioned here that both neat blend component form homogeneous, dense membranes. It is evident that as the weight fraction of P(VBCImBF_4_-co-AA_10_) component is increased, the domain size of the dispersed P(VBCImBF_4_-co-AA_10_) phase is also increased. Similar morphologies were observed for immiscible PI/PPO polymer blends as well [41].

**Figure 7:**
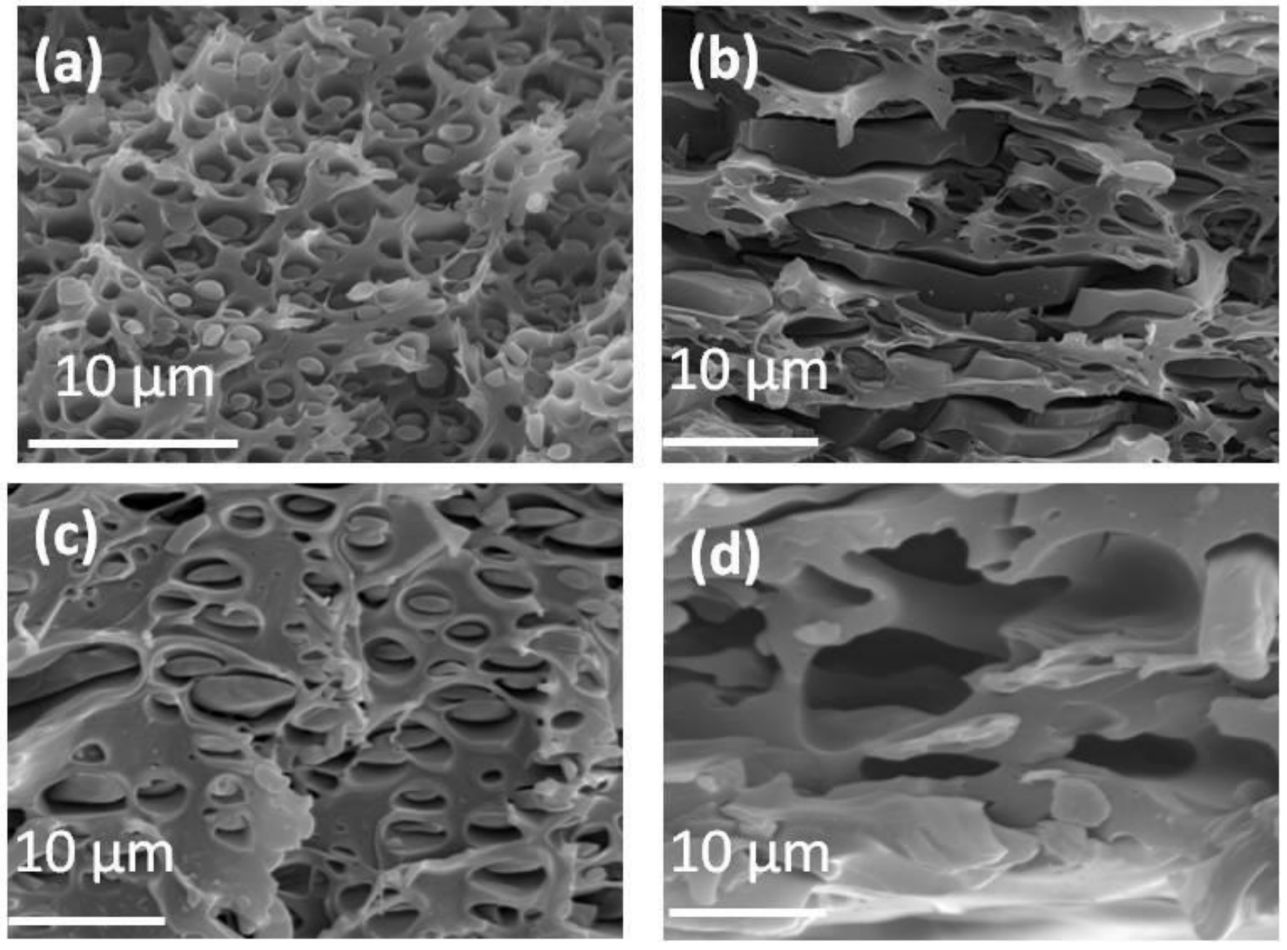
SEM micrographs of blend membranes (**a**) PILPyr-TFSI/P(VBCImTFSI-co-AA_10_) with composition 80/20, (**b**) PILPyr-TFSI/P(VBCImTFSI-co-AA_20_) with composition 80/20, (**c**) PILPyr-TFSI/P(VBCImTFSI-co-AA_20_) with composition 70/30 and (**d**) PILPyr-TFSI/P(VBCImTFSI-co-AA_20_) with composition 60/40.

In the case of PILPyr-TFSI/P(VBCImTFSI-co-AA_10_) with composition 80/20 whereas both blend components contain the TFSI anion, a matrix-droplet morphology is observed, where P(VBCImTFSI-co-AA_10_) droplets are embedded in a continuous PILPyr rich phase. Most of the dispersed domains have diameters ranging from 1–1.5 μm (Figure 7a). As the acrylic acid loading is increased to 20 %, the blend with the same 80/20 composition exhibits a different morphology (a lamellar like) and when the P(VBCImTFSI-co-AA_20_) content is increased to 30% (blend composition 70/30), spherical nodules with a large size distribution are dispersed in the continuous PILPyr rich phase (Figure 7b,c). The diameters of the dispersed nodules varied from 2.6 to 6.7 μm. This nodular morphology has also been observed in immiscible Polyamide 11/poly(hydroxyl amino ether) blends for PA11 contents higher than 50 wt% in the blends [42]. Moreover, further increase of P(VBCImTFSI-co-AA_20_) loading (40 wt%), leads to formation of void with dimensions up to 10 μm probably due to the lack of miscibility between the two components (Figure 7d). 

Regarding the study of the gas separation properties of blend membranes, the imidazolium based PIL could not be prepared as a robust membrane, thus gas permeability measurements of the neat component were not conducted. Nevertheless, a CO_2_ permeability of 9 Barrer and selectivity of 39 for the CO_2_/CH_4_ gas pair for similar PILs based on methyl-imidazolium tethered to a polystyrene backbone has been reported [43]. The second component, PILPyr, can be prepared as a neat membrane and its CO_2_ and CH_4_ permeability values were reported by our group in a previous work [10]. Table 2 presents the permeability and ideal selectivity data for the prepared blend membranes. The effect of blend composition on gas transport properties for PILPyr-BF_4_/P(VBCImBF_4_-co-AA_10_) blends was studied. In particular, neat PILPyr-BF_4_ membrane has a very low CO_2_ permeability (2 Barrer). In the case of blends, as the P(VBCImBF_4_-co-AA_10_) content is increased from 20 to 30%, CO_2_ permeability is also increased from 1.97 to 2.78 Barrer (41% increment), while, on the contrary, CH_4_ permeability was much reduced from 0.041 to 0.018 Barrer (44% reduction). Consequently, the selectivity for CO_2_/CH_4_ gas pair shows a very significant increase from 77 to 154 for blend 70/30, corresponding to 100% increment. Generally, the low CO_2_ and CH_4_ permeability for the neat PILPyr-BF_4_ and its corresponding blends (80/ 20 and 70/30) can be related to their high T_g_ values due to the limited mobility of polymeric chains for gas diffusion [44].

Regarding the effect of counter anion containing in blends on gas permeability, the results of this study further confirm the literature data. In particular, CO_2_ permeability of PILPyr-TFSI/P(VBCImTFSI-co-AA_10_) blend membrane with composition 80/20 (TFSI is the counter anion of both PIL components), increases from 1.97 to 6.47 Barrer (228 % increment) with respect to the 80/20 blend containing BF_4_ as counter anion. This behaviour is attributed to the higher bulkiness of the TFSI anion based on its larger van der Waal’s volume compared to BF_4_ [45], thus enabling a looser chain packing. Interestingly, the selectivity of the blend containing the TFSI anion has only a slight decrease from 77 to 69 when compared to the blend containing the BF_4_^−^ anion. An increase in P(VBCImTFSI-co-AA_10_) content results in an increase of CO_2_ and CH_4_ permeabilities (7.37 and 0.2 Barrer, respectively) of the resultant blend (70/30 composition). In contrast, gas selectivity is decreased from 69 to 37.

The effect of the molar percentage of acrylic acid units on the gas transport properties was also evaluated. The comparison of the permeability data of blends with the same composition and containing the same counter anion, but having 10% and 20% acrylic acid units revealed that blend PILPyr-TFSI/P(VBCImTFSI-co-AA_20_) 80/20 with the increased percentage shows a moderate increase in CO_2_ permeability from 6.47 to 7.00 Barrer (8% increment). However, the simultaneous, sharp increase of selectivity from 69 to 103, corresponding to 48% increment, is an important finding. It is speculated that carboxylic acid groups of acrylic acid participate in mild ionic cross-linking, thus leading to a tighter polymer chain packing and consequently to enhanced selectivity.

Further increase of the P(VBCImTFSI-co-AA_20_) content leads to a sharp increase of both CO_2_ and CH_4_ permeabilities, while the selectivity is abruptly decreased from 102 to 2 for the blend with composition 60/40. The increased permeability is related to the existence of voids (pores) due to the lack of miscibility, as was also evidenced by SEM microscopy (Figure 7d). Gas transport in homogeneous, dense membranes is governed by solution-diffusion mechanism, however, in this case, mass transfer is also facilitated by Knudsen diffusion and viscous flow that take place in porous membranes [46] causing a major drop of selectivity.

For phase separated blends, blend permeability is strongly dependent on the morphology of the two components (one is the lower permeability and the other is the higher permeability component) and can be described by various models such as series, parallel or Maxwell [13,47,48]. These models offer reasonable predictions where one phase is entirely the continuous phase at both ends of the composition range. It would be very useful to compare the experimental gas permeability values with those predicted by the models in order to understand the effect of morphology on gas transport properties. However, blend permeability using the different models can be calculated from the volume fraction and the permeability of each blend component. In our case, since the permeability of the neat imidazolium based PIL cannot be measured, it is not possible to calculate the blend permeability as predicted by the different models.

**Figure 8:**
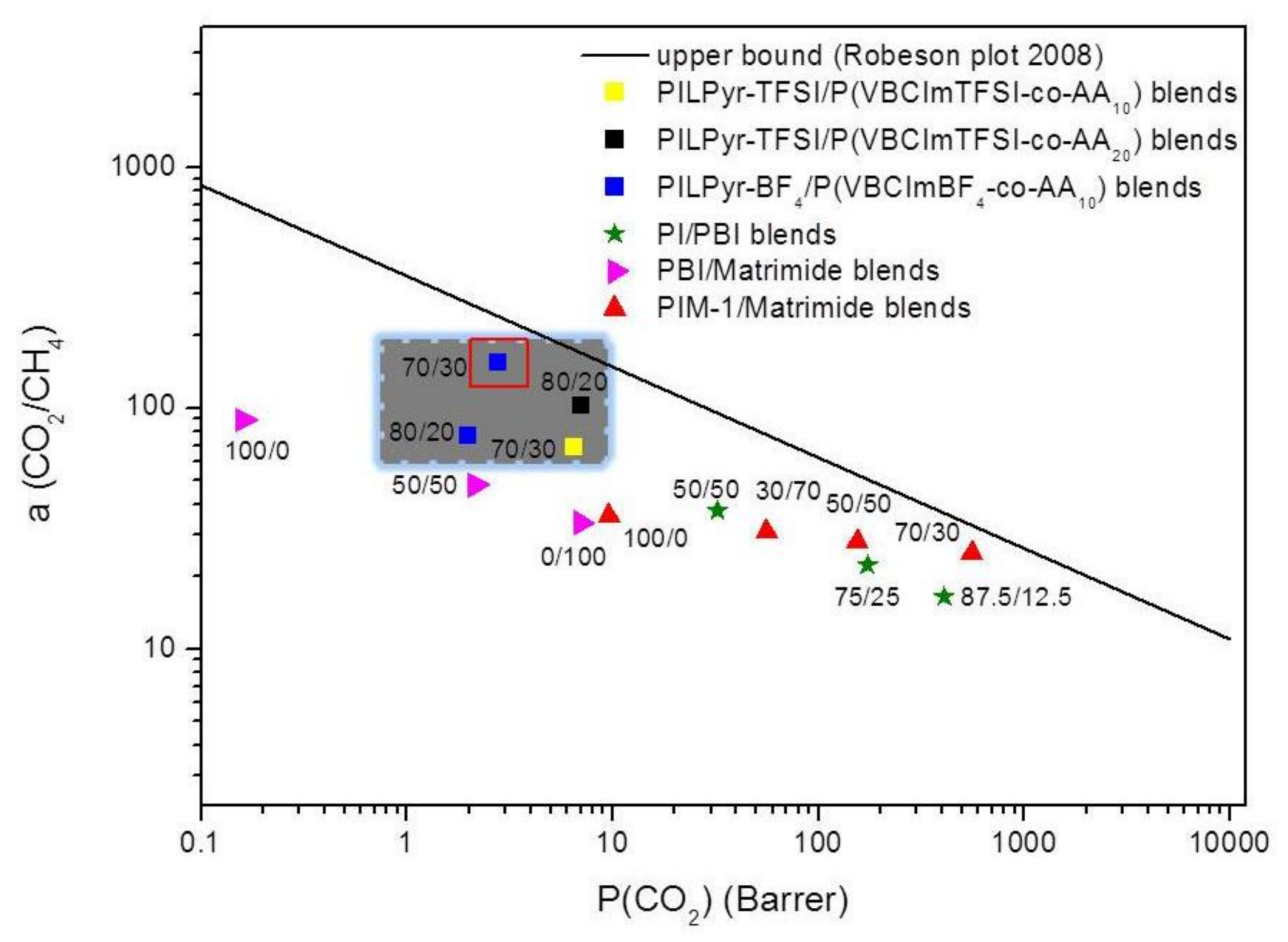
CO_2_/CH_4_ separation performance of prepared blend membranes compared to PI/PBI blends [19], PBI/Matrimid blends [49] and PIM-1/Matrimid blends [50].

The correlation between permeability and selectivity for the CO_2_/CH_4_ gas pair in the form of Robeson plot is shown in Figure 8. Literature data for other immiscible polymer blend membranes are also illustrated in the same plot. The grey highlighted region depicts some of the data of this work. It is evident that the prepared blend membranes lie below the upper bound limit on the left part of the plot characterized by lower permeabilities and higher selectivities compared to other reported membranes. The selectivity in some cases is very high (e.g. 154 for PILPyr-BF_4_/P(VBCImBF_4_-co-AA_10_) with composition 70/30), however, a significant increase in the permeability (to 10^2^–10^4^ Barrer) would be required to compete with membranes at or close to the Robeson upper bound line.

## 4. Conclusions

In conclusion, blend membranes comprised of high molecular weight, thermally stable pyridinium based PIL with excellent film forming ability and imidazolium functionalized poly(vinyl benzyl chloride-co-acrylic acid) backbone PILs have been prepared at different compositions. The effect of blend composition, the type of counter anion as well as the molecular percentage of acrylic acid units on miscibility, microstructure and gas separation properties was investigated. In all cases, thermally stable phase separated blends with varying morphologies were obtained. Regarding the gas separation performance of membranes, in the case of PILPyr-BF_4_/P(VBCImBF_4_-co-AA_10_) blends, by increasing the P(VBCImBF_4_-co-AA_10_) content from 20 to 30%, the CO_2_ permeability is also increased from 1.97 to 2.78 Barrer (41% increment) while the selectivity for the CO_2_/CH_4_ gas pair shows a very significant increase from 77 to 154 (100 % increment) for blend 70/30. Another important finding resulted from the comparison of the permeability data of blends with the same composition and containing the same counter anion (TFSI) but having different percentages of acrylic acid units (10 and 20%). The blend PILPyr-TFSI/P(VBCImTFSI-co-AA_20_) 80/20 with 20% acrylic acid units shows a moderate increase in CO_2_ permeability from 6.47 to 7.00 Barrer (8% increment). Interestingly, CO_2_ permeability was increased and at the same time a very significant increase of the selectivity was also achieved. These membranes could be further developed targeting to CO_2_ permeability enhancement by tuning the imidazolium copolymer structure in order to be used as gas separation membranes. 

## Figures and Tables

**Table 1:** Blend membranes preparation by varying blend composition, counter anion type and molar fraction (*X*) of acrylic acid units contained in P(VBCImY-co-AA*_X_*).

Blend Composition PILPyr-X/P(VBCImY-co-AA_x_)	PILPyr-X	P(VBCImY-co-AA_x_)	
-	X-Counter Anion	Y-Counter Anion	x Molar Fraction of AA Units
PILPyr-BF_4_/ P(VBCImBF_4_-co-AA_10_)			
100/0	BF_4_	-	-
80/20	BF_4_	BF_4_	10
70/30	BF_4_	BF_4_	10
PILPyr-TFSI/ P(VBCImTFSI-co-AA_10_)			
100/0	TFSI	-	-
80/20	TFSI	TFSI	10
70/30	TFSI	TFSI	10
PILPyr-TFSI/ P(VBCImTFSI-co-AA_20_)			
80/20	TFSI	TFSI	20
70/30	TFSI	TFSI	20
60/40	TFSI	TFSI	20

**Table 2:** Single gas permeability as well as ideal selectivity (α) data for the prepared blend membranes.

Polymer Blends	Composition	P_CO2_ (Barrer)	P_CH4_ (Barrer)	*α*CO_2_/CH_4_
PILPyr-BF_4_ /(VBCImBF_4_-co-AA_10_)	100/0	2.0 ± 0.20 ^a^	0.040 ± 0.01 ^a^	50 ± 5
PILPyr-BF_4_/P(VBCImBF_4_-co-AA_10_)	80/20	1.97 ± 0.25	0.041 ± 0.0002	77 ± 2
PILPyr-BF_4_/P(VBCImBF_4_-co-AA_10_)	70/30	2.78 ± 0.15	0.018 ± 0.0002	154 ± 4
PILPyr-TFSI/P(VBCImBF_4_-co-AA_10_)	100/0	4.1 ± 0.10 ^a^	0.100 ± 0.01 ^a^	41 ± 1
PILPyr-TFSI/P(VBCImTFSI-co-AA_10_)	80/20	6.47 ± 0.04	0.094 ± 0,019	69 ± 5
PILPyr-TFSI/P(VBCImTFSI-co-AA_10_)	70/30	7.37 ± 0.03	0.200 ± 0.014	37 ± 2
PILPyr-TFSI/P(VBCImTFSI-co-AA_20_)	80/20	7.00 ± 0.12	0.068 ± 0.039	103 ± 25
PILPyr-TFSI/P(VBCImTFSI-co-AA_20_)	70/30	8.94 ± 0.59	1.43 ± 0.154	6 ± 1
PILPyr-TFSI/P(VBCImTFSI-co-AA_20_)	60/40	17.84 ± 0.08	8.22 ± 0.260	2 ± 0.1

^a^ data obtained from our previous work [10].

## Supplementary Materials

The following are available online at https://www.mdpi.com/2077-0375/9/12/164/s1, Figure S1: ^1^HNMR spectra of (a) precursor copolymer P(VBC-co-AA), (b) P(VBCImCl-co-AA_20_) based PIL containing Cl anion, (c) P(VBCImBF_4_-co-AA_10_) based PIL containing BF_4_ anion and (d) P(VBCImTFSI-co-AA_20_) based PIL containing TFSI anion, Figure S2: ^19^F NMR spectra of (a) P(VBCImBF_4_-co-AA_10_) and (b) P(VBCImTFSI-co-AA_20_), Figure S3: TGA curves of precursor P(VBC-co-AA_10_) and its PILs analogues containing different counter anions, Figure S4: DSC thermograms of PILPyr-BF_4_/P(VBCImBF_4_-co-AA*_10_*) blends, Figure S5: TGA curves of PILPyr-TFSI/P(VBCImTFSI-co-AA_10_) blends, Figure S6: ATR-FT-IR spectra of PILPyr-TFSI/P(VBCImTFSI-co-AA*_20_*) blends.
