# Supplementary material for: Synthesis of Imidazolium based PILs and Investigation of Their Blend Membranes for Gas Separation
Source: Membranes (Basel). 2019 Dec 3;9(12):164. doi: 10.3390/membranes9120164 (PMC6950310; doi:10.3390/membranes9120164)
Supplement: Supplementary file 1 [file membranes-09-00164-s001.pdf]

membranes

MDPI

Supplementary Materials: Synthesis of imidazolium based PILs and investigation of their blend membranes for gas separation

Thanasis Chouliaras [1], Aristofanis Vollas [1], Theophilos Ioannides [2], Valadoula Deimede [1,*] and Joannis Kallitsis [1,2]

Commented [M1]: Please carefully check the accuracy of names and affiliations. Changes will not be possible after proofreading.

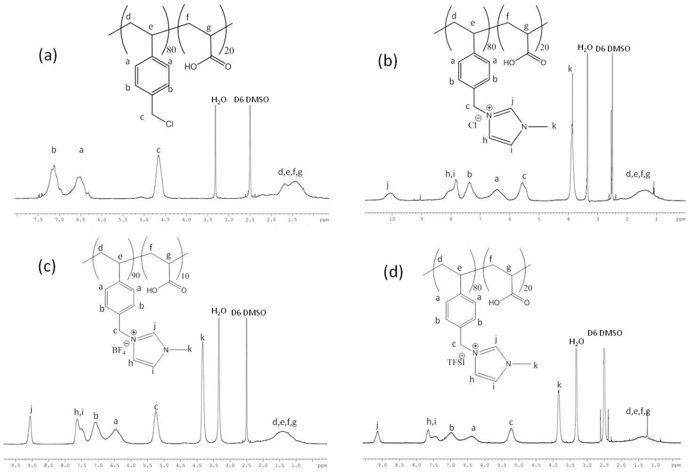

Figure 1. ^{1}HNMR spectra of (a) precursor copolymer P(VBC-co-AA), (b) P(VBCImCl-co-AA$_{20}$) based PIL containing Cl anion, (c) P(VBCImBF$_4$-co-AA$_{10}$) based PIL containing BF$_4$ anion and (d) P(VBCImTFSI-co-AA$_{20}$) based PIL containing TFSI anion.

^{1}HNMR spectroscopy measurements were also employed in order to confirm anion exchange in the synthesized PILs (Figure S1). It is evident that for PILs containing either BF$_4$ or TFSI anions, peaks assigned to protons of imidazolium ring as well as aromatic protons of VBC backbone are shifted upfield when compared to PIL containing chloride anions. For example, the slightly acidic proton at C2 position in imidazolium ring is more shielded and shifted from 10.16 ppm (**j** in Figure S1) to 9.1 ppm when the anion was exchanged from chloride to BF$_4$ or TFSI, as a result of the stronger interaction e.g. hydrogen bonding between the chloride anion and C-2 position proton. These findings together with ^{19}FNMR data (Figure S2) support that successful anion exchange reactions took place.

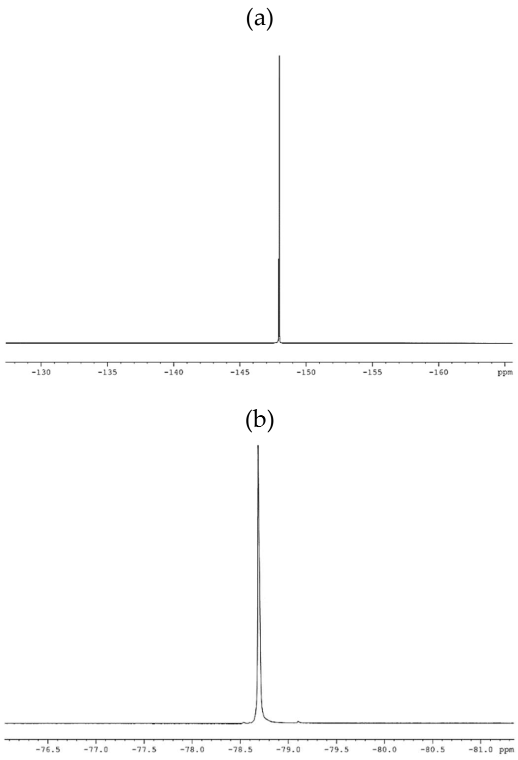

Figure 2. ^{19}F NMR spectra of (a) P(VBCImBF4-co-AA10) and (b) P(VBCImTFSI-co-AA20).

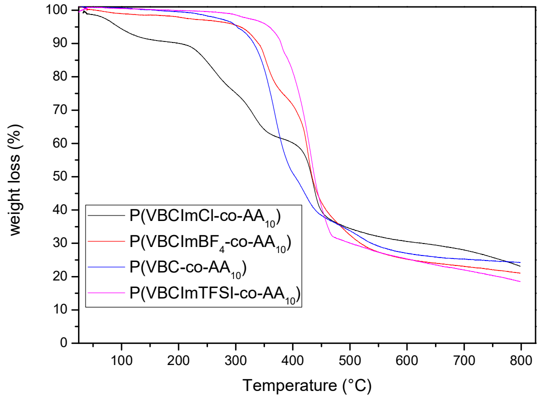

Figure 3. TGA curves of precursor P(VBC-co-AA10) and its PILs analogues containing different counter anions.

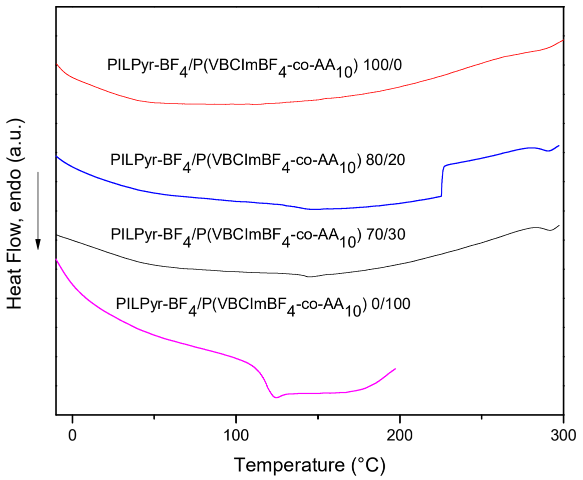

Figure 4. DSC thermograms of PILPyr-BF_4/P(VBCImBF_4-co-AA_{10}) blends.

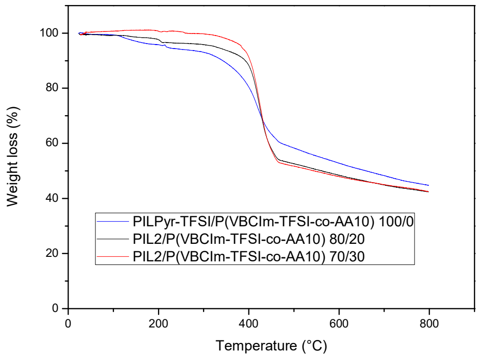

Figure 5. TGA curves of PILPyr-TFSI/P(VBCImTFSI-co-AA_{10}) blends.

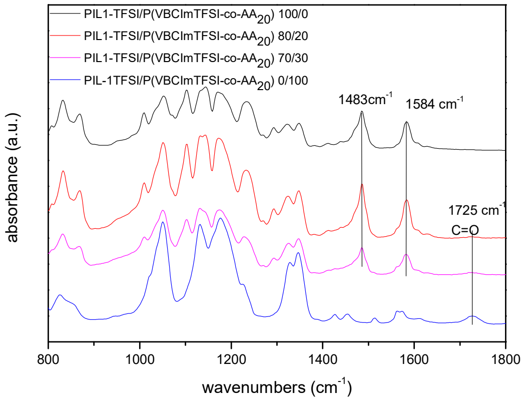

Figure 6. ATR–FT–IR spectra of PILPyr-TFSI/P(VBCImTFSI-co-AA20) blends.

© 2019 by the authors. Submitted for possible open access publication under the terms and conditions of the Creative Commons Attribution (CC BY) license (http://creativecommons.org/licenses/by/4.0/).
